# Senescent cell-derived vaccines: a new concept towards an immune response against cancer and aging?

**DOI:** 10.18632/aging.205975

**Published:** 2024-06-26

**Authors:** João Pessoa, Sandrina Nóbrega-Pereira, Bruno Bernardes de Jesus

**Affiliations:** 1 Department of Medical Sciences and Institute of Biomedicine - iBiMED, University of Aveiro, Aveiro 3810-193, Portugal

**Keywords:** cancer, immunotherapy, tumor-associated senescent cells, senescence, antigen, vaccine

## Abstract

Two recent seminal works have untangled the intricate role of tumor-associated senescent cells in cancer progression, or regression, by guiding our immune system against cancer cells. The characterization of these unique, yet diverse cell populations, should be considered, particularly when contemplating the use of senolytics, which are drugs that selectively eliminate senescent cells, in a cancer framework. Here, we will describe the current knowledge in this field. In particular, we will discuss how the presence of senescent cells in tumors could be used as a therapeutic target in immunogenic cancers and how we may hypothetically design an adaptive anti-aging vaccine.

## INTRODUCTION

Senescent cells have been widely characterized [1–5]. Different research lines have extensively demonstrated that senescent cells accumulate in different tissues during aging, where they guide the aging process through local and systemic signals. While aging is characterized by an accumulation of senescent cells associated with a loss of tissue fitness, senescence per se is a strong barrier to tumor progression [6–8]. These findings hold exciting promise in the combat of both cancer and aging. In fact, expression of tumor-suppressor factors (Sp53/Sp16/SArf), was shown per se to increase the lifespan of rodents, supporting the hypothesis that the benefits of senescence may outweigh the deleterious effects of its accumulation later in age, at least in small rodents [9, 10]. This may relate to a protective background in a cancer-prone model such as the WT C57BL/6 mice in ad libitum diet [11].

Induction of senescence, triggered by internal or external signals, results in a committed cell cycle arrest. Oncogenic induced senescence (OIS), originally observed as a strong barrier against cancer (and initially described after H-RasG12V expression in IMR-90 cells), presents sustained expression of p16 and p53 activation, something also observed during replicative induced senescence [7, 12–14].

Although one could not distinguish senescent tumor cells (STC) from non-tumoral senescent cells, it is suggested that STC are derived during cancer progression. Furthermore, current cancer treatments could generate senescent cells in the tumor vicinity. Radiotherapy, or other tumor-targeted therapies, have been shown to induce senescence in the tissues surrounding tumors, which have the capacity to fuel cancer progression [15] through mechanisms further explored in this review. Rather than senescent cells originated directly from tumor cells, the myriad of cell states around or within the tumor microenvironment is the guiding force of tumor-associated senescent cells (TASCs). Hereafter, we will use the term TASCs to refer to the different types of senescent cells coexisting in the core tumor and its vicinity.

How TASCs could be used to design novel anticancer strategies was recently addressed [16, 17]. Here, we will discuss the latest advances on our knowledge concerning the capacity of senescent cells to guide the immune system towards different types of cancer. Furthermore, we will integrate this information with new anti-aging senolytic-based strategies.

## TASCs have both tumor-suppressive and oncogenic impacts

Senescence is evolutionarily regarded as a developmental anti-cancer program. However, this unique perception of senescence has been challenged by our improved understanding of the intricate role of senescent cells during the aging process. Depending on their background, TASCs can promote tumor growth and aggressiveness, or function as a strong barrier to cancer progression. DNA damage, oncogene activation and mutations in tumor suppressor genes may induce TASCs to prevent tumor formation [7, 18–20]. For instance, senescence induced by telomere dysfunction and oncogenic signaling (and further accelerated by oncogene-induced DNA replication stress) is a biological response of cells from human cancer precursor lesions and may work as a tumor-suppressive mechanism through cell cycle arrest [3, 21].

As opposed to their tumor-suppressive effects, TASCs may also have an oncogenic impact. The senescence-associated secretory phenotype (SASP) is an intercellular strategy employed by senescent cells to communicate with the surrounding tissues and to signal the senescence process, through the release of chemokines, cytokines, growth factors and enzymes [22]. Cellular stressors can activate the DNA damage response, which, when persistent, can induce transcription factors and signaling pathways that trigger SASP release and affect its composition [23, 24]. The SASP is regulated at the messenger RNA transcription, stability, translation, and secretion levels [24]. SASP components include cytokines (such as interleukin-6 [IL-6], tumor necrosis factor α, tissue growth factor β, and others), chemokines (such as C-C motif ligand 2 and C-X-C motif ligand 2), bioactive lipids, reactive oxygen species (ROS), and noncoding DNA or RNA molecules [23]. In 30-70% senescent cells, the SASP contains molecules with pro-inflammatory, pro-apoptotic, and pro-fibrotic effects. In the remaining 70-30% senescent cells, the SASP is thought to contain growth and regenerative factors, which can possibly reduce the levels of apoptosis, fibrosis, and tissue destruction [23]. The SASP can also amplify its signals through autocrine and paracrine positive feedback loops [24]. Moreover, some SASP components can propagate senescence properties to neighboring or distant non-senescent cells [23].

Evidence suggests that the SASP could lead to an inflammatory microenvironment supporting tumor progression and drug extrusion causing chemotherapy resistance. In these processes, IL-6, C-X-C motif chemokine ligand 10 and metaloproteinases 3/9 play pivotal roles [7, 25–31]. Supporting the oncogenic impact of TASCs, senescence was shown to support melanoma progression [32]. Furthermore, the increase of senescence and the inflammatory microenvironment triggered by the SASP was shown to facilitate renal cell carcinoma metastasis [33]. In mice, senescent dermal fibroblasts generated a SASP signature that promoted carcinoma growth after co-transplantation. Importantly, this response was observed only in immune-competent mice demonstrating the critical role of the immune system (in particular through IL-6) in the spreading of senescence signaling [29, 34].

The presence of senescent cells in cancer has been widely characterized. A body of preclinical studies globally demonstrates that cancer therapies (e.g. radiotherapy or chemotherapy) cause the accumulation of senescent cells, both in tumors (TASCs) and in the surrounding healthy tissues of the cancer patient. Because of the dual effect of senescent cells in cancer therapy, a two-step therapeutic approach has been proposed, in which radiation and chemotherapy induce senescence (with anticancer effects) and, subsequently, senescent cells are selectively cleared, to minimize tumor fueling [35]. These studies suggest that, for optimized cancer treatment, senescence should be transiently induced and subsequently eliminated.

Strategies to tackle senescent cells have been widely described [36, 37]. In this framework, the use of senolytics, which are compounds that specifically eliminate senescent cells by inducing their apoptosis, holds great promise in enhancing the effectiveness of cancer therapy and minimizing its side effects [38]. Senolytics are broadly divided into first and second-generation. First-generation senolytics are natural products with multiple molecular targets and include the anticancer agent dasatinib and the natural flavonoid quercetin. These compounds target ephrins 1/3 and phosphatidylinositol 3-kinase, respectively, which are critical for senescent cell survival [39]. Clinical trials in aging-related diseases, including heart, kidney, liver, muscular, and neurological diseases, have shown that the combined effects of these two compounds killed senescent cells [23]. Second-generation senolytics include compounds and nanoparticles activated through contact with the increased lysosomal content or increased senescence-associated β-galactosidase activity of senescent cells. Other second-generation senolytics include SASP inhibitors and inducers of sodium-potassium pump-dependent apoptosis [23]. Since senescence phenotypes are heterogeneous, senolytic effects are also heterogeneous, depending on the cell type and the mechanism that triggered senescence. Therefore, the simultaneous use of more than one senolytic has been tested to tackle senescent cell heterogeneity [39]. Senolytic mechanisms include the triggering of the intrinsic pathway of apoptosis, by increasing p53 release or targeting anti-apoptotic members of the B-cell lymphoma protein 2 family. Other senolytic mechanisms include the simultaneous inhibition of multiple cellular pathways or the depletion of metabolites that are essential for senescent cell survival. Additional mechanisms include the aggravation of cellular stresses, such as destabilization of proteostasis, accumulation of ROS, or mitochondrial dysfunction. Since senescent cells are already under stress, these strategies can promote their selective elimination [39].

In a cancer framework, Patil and colleagues recently demonstrated that treatment of irradiated mice with a small molecule that tackles senescent cells (the senolytic agent ABT-737) was sufficient to reduce radiation-enhanced tumor growth through the control of the expression of 12-lipoxygenase (12-LOX), a molecule mediating the deleterious effects of senescence. These data demonstrate how radiation-induced senescence could support tumor growth, and identified a key component (12-LOX) of the oncogenic microenvironment [40]. Another compound, ABT-263, has demonstrated powerful senolytic activity against senescent breast cancer cells generated by radiation or chemotherapy [41]. Of note, several senolytic compounds are currently under clinical trials [42]. Moreover, senolytics are also promising in overcoming the decline in the immune fitness of the elderly, which is partially caused by immune cell senescence [43].

## TASCs and other senescent cells share cancer antigens

Marin et al. and Chen et al. have recently demonstrated how senescent cells could be used to guide the immune system against cancer [16, 17]. In fact, previous evidence demonstrated that TASCs could lead to the elimination of established tumors when injected into tumor-bearing mice, through the mobilization of an antitumoral cytotoxic T lymphocytes response [44]. This observation has been strengthened by recent studies. In cell lines derived from mouse models of melanoma and pancreatic carcinoma, TASCs were induced through the conjunction of radiotherapy and inhibition of DNA repair. These senescent cells were shown to express immunostimulatory cytokines that could activate cytotoxic T lymphocytes. When TASCs were injected into tumor-bearing mouse models, an antitumor immune response was generated, indicating that vaccination with senescent cells could halt tumor growth [17]. Using a p53-restorable liver cancer model, Chen and colleagues corroborated previous findings and unveiled the paradoxical effect of senescent cells on tumor biology [16]. In their model, a combination of different SASP programs, in conjunction with differential IFNγ outputs, was shown to influence the oncogenic and tumor-suppressive dual effect of senescence in (liver) cancer. In fact, different SASP programs may predict patient outcomes in ovarian and breast cancer through a differential modulation of a senescence program [45, 46]. These studies have improved our understanding of the impact of TASCs on cancer regression and potential relapse.

TASCs may also help training the immune system against cancer, revealing their adjuvant capacity. Liu and colleagues demonstrated that mouse colon and mammary carcinoma cell lines treated with radiation and veliparib to generate cancer senescent cells could induce the maturation of co-cultured dendritic cells, leading to efficient priming of cytotoxic T lymphocytes. Injecting these TASCs into tumor-bearing mice increased inflammation and suppressed tumor growth, enhancing the radiotherapy effect and blocking colonization by tumor cells [47]. TASCs could source a combination of immunostimulatory and immunosuppressive molecules, whose equilibrium may critically affect the outcome of immunotherapy [48]. MHC class I overexpression on TASCs depends on paracrine type I interferon signaling. TASCs are naturally antigenic, displaying antigens that can be targeted by CD8-positive T cells. Importantly, some of these antigens are exclusive of senescent cells.

Senescent cells can also release damage-associated molecular patterns, including ATP, at levels comparable to cells undergoing immunogenic cell death, being able to efficiently transfer antigens to dendritic cells. This ability suggests that inducing senescence of tumor cells would significantly enhance their adjuvanticity. Senescent melanoma and pancreatic cancer cells transplanted into immunocompetent mice prevented tumor development upon re-inoculation with identical proliferating cancer cell types. Nevertheless, when tumors were already established, inoculation with senescent cancer cells could delay, but not fully stop, tumor progression. Moreover, the induction of senescence in four distinct patient-derived primary cancer cells lines resulted in high levels of stimulation of their own tumor-infiltrating lymphocytes, with anticancer effects by enhancing the immunogenicity of these cancer cells [17, 48]. These effects were critically mediated by the enrichment of TASCs and senescent cells in unique peptides, which were able to activate strong immune responses.

The above-mentioned studies demonstrate that TASCs could effectively repurpose the immune system against cancer cells. Therefore, it is likely that TASCs can also guide the immune system against other types of senescent cells, with a potential impact on the aging process.

## Cancer immunotherapy could also be anti-aging

Senescent cells are directly related to aging and aging-related diseases. As such, their selective targeting would be an asset for the prevention and treatment of those diseases. Moreover, as discussed in the previous sections, the dual role of TASCs in cancer therapy raises the convenience of their removal after transient exposure to the tumor microenvironment. From these concepts, an immunotherapeutic approach for the selective elimination of senescent cells would be convenient.

Currently available immunotherapeutic approaches are generally not indicated to be selective for TASCs. For example, many immunotherapy approaches are available for breast cancer [49] including different subtypes such as triple-negative breast cancer [50]. None of those immunotherapy approaches seems to have been designed to specifically target TASCs, which have shown increased resistance to immunotherapy [51]. Whether cancer immunotherapy approaches can selectively target senescent cells is an aspect that is generally poorly discussed in the literature. Interestingly, two of the most valuable immunotherapy targets, programmed death-ligand 1 (PD-L1) and cytotoxic T lymphocyte antigen 4 (CTLA4) [52], are positively correlated with senescence. PD-L1 is upregulated in senescent cells [53], which accumulate with aging and have increased resistance to immune surveillance [54]. Moreover, CTL4 was shown to be upregulated in aged human individuals [55]. As such, immunotherapies targeting PD-L1 or CTL4 may also have some specificity against senescent cells.

A major limitation of cancer immunotherapies is the risk of senescence of their effector cells, which compromises their therapeutic role. The chimeric antigen receptor T (CAR-T) and the T cell receptor-engineered T (TCR-T) cell therapies are two successful immunotherapy approaches that exploit T cells. One of their major limitations is the risk of T cell senescence, for which strategies to reverse senescence have been tested [56]. Senescent T cells have been proposed as potential targets for cancer immunotherapy, through the prevention or reversal of their senescent state [57]. Therefore, the development of senescence-specific immunotherapies would be also beneficial to already available cancer immunotherapies.

Importantly, some strategies are already available to induce the selective elimination of senescent cells. In mice, CD4^+^ T cells were able to target oncogene-induced senescent hepatocytes through MHC-II recognition [58]. Also in mice, natural killer cells could eliminate senescent fibroblasts through the recognition of the NKG2D receptor on their surface [59]. These findings encourage the development of cost-effective senescent cell-specific immunotherapy approaches [42].

## Conclusion

The exploitation of the patient’s immune system to eliminate cancer cells has long been tested through the development of innovative strategies with remarkable success and high expectations [60–66]. Our growing understanding of the immune system has allowed the design of novel anticancer therapies. However, tumor cells are generally poor antigen-presenting cells, evading the immune response in early stages of the pathophysiology [67] and restricting immunotherapeutic efficacy to only a minor group of cancers [68, 69]. Nevertheless, targeting of senescent cells in the context of cancer and aging may upsurge as an alternative to this critical limitation, through the selective activation of a T cell-specific response against senescent cells within the tumor or its vicinity.

**Figure 1:**
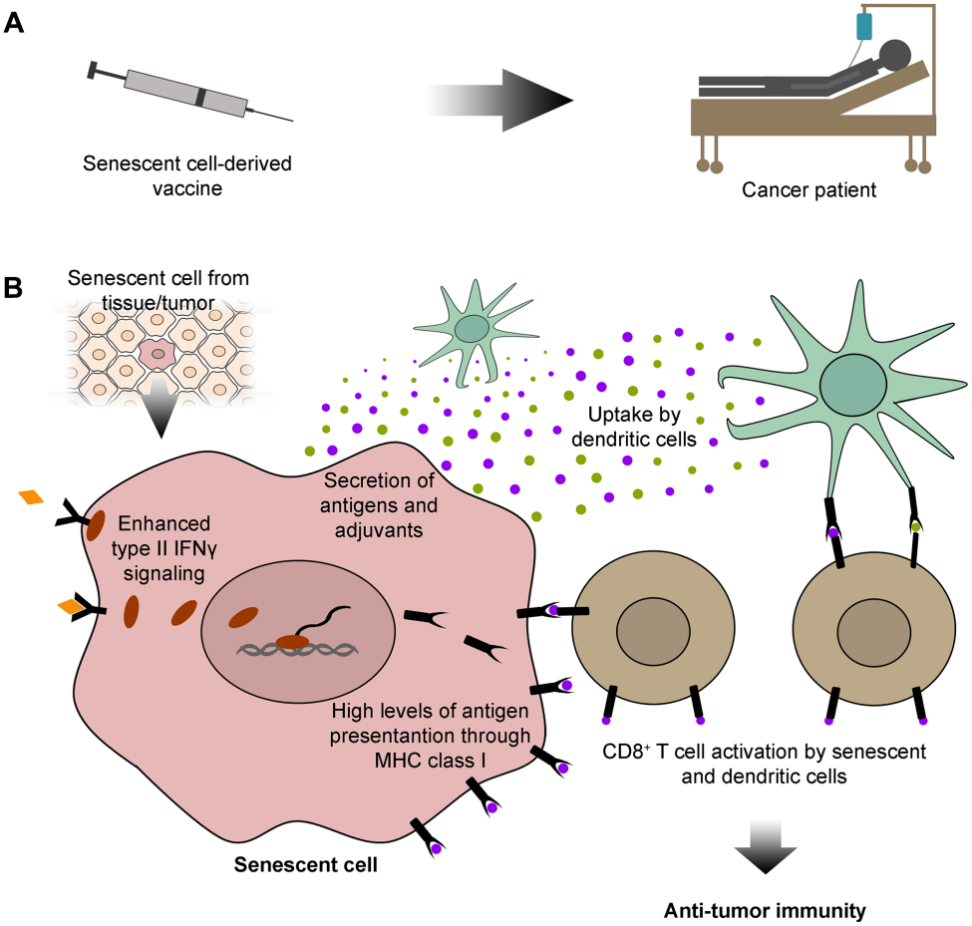
**Senescent cell-derived vaccines: sources of specific antigens for cancer immunotherapy.** (**A**) Here, we propose that senescent cells could be exploited for developing immunotherapeutic vaccines for cancer patients. (**B**) Senescent cells used in the development of these vaccines could be isolated from tissues or tumors. Senescent cells have outstanding features that could be exploited for cancer immunotherapy. These include high levels of type II interferon γ (IFNγ) signaling, which lead to abundant antigen presentation on the cell surface, through the major histocompatibility complex (MHC) class I. Senescent cells also secrete a vast array of antigens and adjuvants, which can be internalized and subsequently displayed by dendritic cells. Both senescent and dendritic cells can further activate CD8^+^ T cells with antigens common to senescent and cancer cells, empowering CD8^+^ T cells with an anti-tumor immune capacity.

Recent evidence supports the use of TASCs as sources of peptide antigens and adjuvants for anticancer vaccine development (Figure 1A). Their SASP provides abundant release of stimulatory cytokines, which, in conjunction with high levels of antigen presentation, generates a robust tumor specific T cell response (Figure 1B). As discussed here, this approach will potentiate their adjuvanticity in cancer targeting, allowing the design of stronger and directed immunotherapeutic strategies. Moreover, since cancer and senescent cells share common antigens, this immunotherapeutic approach could also be effective against aging and age-related diseases. Therefore, cancer immunotherapy based on TASCs and other types of senescent cells may achieve exciting outcomes beyond cancer therapy.
